# Novel Combination of Prebiotics Galacto-Oligosaccharides and Inulin-Inhibited Aberrant Crypt Foci Formation and Biomarkers of Colon Cancer in Wistar Rats

**DOI:** 10.3390/nu8080465

**Published:** 2016-08-01

**Authors:** Tahir Rasool Qamar, Fatima Syed, Muhammad Nasir, Habib Rehman, Muhammad Nauman Zahid, Rui Hai Liu, Sanaullah Iqbal

**Affiliations:** 1 Department of Food Science and Human Nutrition, University of Veterinary & Animal Sciences, Lahore 54000, Punjab, Pakistan; tahirnutritionist@gmail.com (T.R.Q.); syed199146@gmail.com (F.S.); nasir@uvas.edu.pk (M.N.); 2 Department of Physiology, University of Veterinary & Animal Sciences, Lahore 54000, Punjab, Pakistan; habibrehman@uvas.edu.pk; 3 Department of Population Medicine and Diagnostic Sciences, Cornell University, Ithaca, NY 14850, USA; mnz9@cornell.edu; 4 Department of Food Science, Cornell University, Ithaca, NY 14850, USA; rl23@cornell.edu

**Keywords:** prebiotics, galacto-oligosaccharides, inulin, biomarkers, colon cancer

## Abstract

The selectivity and beneficial effects of prebiotics are mainly dependent on composition and glycosidic linkage among monosaccharide units. This is the first study to use prebiotic galacto-oligosaccharides (GOS) that contains β-1,6 and β-1,3 glycosidic linkages and the novel combination of GOS and inulin in cancer prevention. The objective of the present study is to explore the role of novel GOS and inulin against various biomarkers of colorectal cancer (CRC) and the incidence of aberrant crypt foci (ACF) in a 1,2-dimethyl hydrazine dihydrochloride (DMH)-induced rodent model. Prebiotic treatments of combined GOS and inulin (57 mg each), as well as individual doses (GOS: 76–151 mg; inulin 114 mg), were given to DMH-treated animals for 16 weeks. Our data reveal the significant preventive effect of the GOS and inulin combination against the development of CRC. It was observed that inhibition of ACF formation (55.8%) was significantly (*p* ≤ 0.05) higher using the GOS and inulin combination than GOS (41.4%) and inulin (51.2%) treatments alone. This combination also rendered better results on short-chain fatty acids (SCFA) and bacterial enzymatic activities. Dose-dependent effects of prebiotic treatments were also observed on cecum and fecal bacterial enzymes and on SCFA. Thus, this study demonstrated that novel combination of GOS and inulin exhibited stronger preventive activity than their individual treatments alone, and can be a promising strategy for CRC chemoprevention.

## 1. Introduction

Colorectal cancer (CRC) is one of the major causes of mortality in both genders among cancer-related deaths worldwide. Being quite a complex process, many factors contribute to the onset of colon cancer and the major risk factors include family history, age, pre-carcinogens present in the food chain and environment [1,2], inflammatory bowel diseases, low intake of vegetables, fruits and fibers, high consumption of red meat and processed meat [3,4], as well as hereditary genetic factors [5]. Epidemiological studies suggest that high intake of fruits and vegetables in human diets has been linked to a lower risk of colon cancer. Excessive scientific and public concerns have been shown in earlier studies to identify naturally-occurring substances in food for CRC chemoprevention [6,7]. In chemoprevention, those natural or synthetic bioactive compounds are used to prevent, delay, or reverse the formation of adenomas, as well as their progression into carcinomas through signal transduction pathways in tumor cells. Recently, prebiotics have gained much attention as natural dietary ingredients which have the potential to maintain a healthy environment in gastrointestinal tract to improve intestinal functions and to prevent colon cancer [8].

Prebiotics are emerging as bioactive ingredients in foods and can positively influence the gastrointestinal microbiota and metabolism [9]. The beneficial bacteria residing in the colon causes fermentation of prebiotics to produce short chain fatty acids (SCFA), including acetate, propionate, and butyrate, which are further involved in prevention of CRC [10,11]. Prebiotic galacto-oligosaccharides (GOS) are produced through transgalactosylation of lactose catalyzed by β-galactosidase (EC 3.2.1.23). During this process various products may be produced having β-1,3, β-1,4, or β-1,6 glycosidic linkages depending upon the source of the enzyme [12]. Previous studies have demonstrated that GOS having different glycosidic linkages have different health effects on the host [13], so glycosidic linkages used between monosaccharide residues is of high importance in imparting health benefits.

The current study was performed to assess, for the first time, the combined effects of prebiotics GOS and inulin, as well as their individual effect on the various biomarkers of the initiation process of rat colon carcinogenesis. Several previously-performed experimental studies have shown that administration of prebiotics, probiotics [14], and synbiotics [15] provide protective and preventive effects against early biomarkers and tumor development in the colon of carcinogen-induced rats [16,17,18]. Research on GOS’ role in colon cancer prevention is very limited to date. GOS used in previous studies had β-1,4 as the primary glycosidic linkage [19,20] and no product with β-1,3 or β-1,6 linkages have yet been used against any cancer treatment. To the best of our knowledge, this is the first time to use prebiotic GOS that contains β-1,6 and β-1,3 glycosidic linkages in a cancer prevention study. 

Inulin-type prebiotics have been extensively studied for their potential benefits, and a large number of experimental studies have shown anti-carcinogenic effects of inulin this is the reason we added inulin as the positive control in our experiment to compare GOS anti-carcinogenic effects with inulin, as very limited data is available for the efficacy of GOS against CRC. No study is available on the combined effect of GOS and inulin against CRC prevention. The objectives of the present study were to investigate the preventive effects of inulin and GOS supplementation separately and in combination against the incidence of aberrant crypt foci (ACF) in a 1,2-dimethyl hydrazine dihydrochloride (DMH)-induced rodent model and to evaluate their effects on various biomarkers of colon cancer. Our study suggests that GOS had a dose-dependent preventive effect on biomarkers of colon cancer and the combined effect of GOS and inulin exhibited stronger preventive activity than their individual treatments alone.

## 2. Materials and Methods

### 2.1. Chemicals

All chemicals and reagents were analytical grade and were purchased form Merck Chemicals (Darmstadt, Germany) unless otherwise stated. The DMH, m-nitrobenzoic acid, phenolphthalein-β-d-glucuronide, nitrophenyl-β-d-glucoside, and *ortho*-nitrophenyl-β-d-galactopyranoside (*o*NPG) were purchased from Sigma-Aldrich (St. Louis, MO, USA). The prebiotic inulin extracted from chicory roots was purchased from Cargill^®^ (Minneapolis, MN, USA).

### 2.2. Animals

Six-week old male Wister rats were purchased from the University of Agriculture, Faisalabad-Pakistan and were housed in temperature and humidity control room (22 ± 2 °C and 55% ± 10%) under 12 h light and dark cycle. All rats received basal diet for one week acclimatization period before beginning the actual experiment. The experimental protocols used herein were approved by the University Ethics Committee for Animal Research. 

### 2.3. Experimental Design

Rats were randomly divided into seven groups (12 per group); G1 was control group fed on AIN-93G/M as basal diet [21]. G2 (DMH alone) was DMH control group fed on basal diet, and Groups G3–G7 were treatment groups. Treatments with prebiotic GOS were given to Groups G3–G5. Group G6 was given inulin and Group G7 received combination of GOS and inulin along with basal diet. Dose was calculated using the human equivalent dose (HED) equation: HED = animal dose in mg/kg × (animal weight in kg/human weight in kg)^0.33^ [22]. Groups G3, G4, and G5 received 76 mg (HED = 4 g), 114 mg (HED = 6 g), and 151 mg (HED = 8 g) GOS, respectively. Group G6 received 114 mg (HED = 6 g) of inulin and group G7 was given combination of GOS and inulin (GOS 57 mg + inulin 57 mg) 114 mg (HED = 6 g). The doses of prebiotics were given orally through tube feeding for a period of 16 weeks according to each group’s mean body weight at beginning of experiment and were adjusted at the end of each week according to body weight changes. In order to make experimental conditions similar, groups G1 and G2 were administered the same amount of water orally. After 16 weeks, all animals were sacrificed by injection of 45 mg/kg body weight of sodium pentobarbital anesthesia.

### 2.4. Carcinogenic Injection

After acclimatization period of one week, respective groups were given prebiotics daily and after completion of four weeks of prebiotics doses, groups G2–G7 received four subcutaneous injections of DMH, 40 mg/kg body weight, twice a week for 2 weeks [23,24]. While G1, control group received similar subcutaneous injections of Ethylenediaminetetraacetic acid (EDTA) solution of pH 6.0 (DMH vehicle). All prebiotic doses were continued during DMH administration.

### 2.5. Preparation of Prebiotic GOS

The *Escherichia coli* BL21 (DE3) containing β-galactosidase (β-gal) gene from *Lactobacillus reuteri* L103 was courtesy of Dietmar Haltrich, Food Biotechnology Laboratory, University of Natural Resources and Life Sciences, Vienna Austria and was used for β-gal production. The enzyme, β-gal was produced by following the procedure explained by Iqbal et al. [25] and enzyme activity was measured for *o*NPG and lactose. The crude cell extract of β-gal was used for the production of prebiotic GOS through transgalactosylation of lactose (250 g/L) prepared in 50 mM sodium phosphate buffer, pH 6.5 at 37 °C. The reaction was carried out for 5 h and immediately stopped by heating at 95 °C for 5 min and stored at −20 °C for further analysis. The Megazyme assay kits (Wicklow, Ireland) were used to analyze glucose (GOPOD assay kit, K-GLUC), galactose and lactose (Lactose/Galactose assay kit, K-LACGAR) in the transgalactosylated mixture by following standard protocol given in the manual and GOS were calculated by subtraction method. The maximum GOS were produced at 75% lactose conversion after 5 h of reaction and the final mixture contained 30% GOS, 30% d-glucose, 15% d-galactose and 25% untransgalactosylated lactose. Furthermore, the mixture of GOS was composed of mainly disaccharide (allolactose) and tri-saccharides followed by tetra-saccharides. It was also observed that maximum GOS were produced at 5 h of lactose conversion and as the reaction continued, all GOS were converted to monosaccharides, glucose, and galactose.

### 2.6. Measurement of Body Weight Changes

The body weight changes of rats were recorded weekly on weighing scale (Model No. SCL66110 Olympia Plus Kent Scientific Corporation, Torrington, CT, USA).

### 2.7. pH and Ammonia

The pH of cecal and fecal digesta were measured using a microelectrode and a pH/ION meter (Model No. HI 111, Hanna Instruments, Ann Arbor, MI, USA). The ammonia concentration was determined by the method described by Lin and Visek [26].

### 2.8. Aberrant Crypt Foci (ACF) Analysis

After animals were sacrificed, the colon was carefully removed, opened longitudinally, and gently rinsed with saline to remove residual bowel contents followed by fixing flat in 10% buffered formalin for 24 h at room temperature. The colon was divided into proximal (near the cecum), middle, and distal colon (near the rectum). Methylene blue (0.2%) was used to stain all these three sections and ACF counting was performed under light microscope. The total number of ACF per rat were calculated as the sum of small, medium, and large ACF in colon [27].

### 2.9. Samples Collection and Enzyme Analysis

To ensure fresh fecal samples, before sacrificing animals fecal samples were collected by gently squeezing the rectal region of rats and cecum samples were collected after sacrificing each animal, which were processed immediately after collection. Azoreductase activity was determined according to the method described by Goldin and Gorbach [28] and for nitroreductase, β-glucuronidase, β-glucosidase, and azoreductase activities minor modifications were made in methods described by Goldin and Gorbach [28]. 

#### 2.9.1. Nitroreductase Assay

Cold pre-reduced (0.2 M; pH 7.8) Tris-HCl buffer was used to suspend fresh cecum and fecal samples. Specimens were disrupted using spatula and were agitated by adding glass beads of 0.2-mm diameter in a tightly stoppered tube for several minutes on a vortex mixer. The supernatant was collected anaerobically by centrifuging the suspension at 500× *g* for 10 min. This supernatant was further processed for enzyme assay. The reaction was carried out anaerobically for 1 h at 30 °C (pH 7.8). The total volume of reaction mixture was 500 µL containing 0.08 M Tris-HCl buffer, 0.35 mM m-nitrobenzoic acid, 0.5 mM NADPH, 1 mM NADH and 200 µL fecal and cecum extracts. At the end of reaction 750 µL HCl of 1.2 N concentration was added in reaction mixture to stop chemical process. To measure the amount of m-aminobenzoic acid produced, readings were taken at 550 nm. A standard curve was prepared by using the Bratton-Marshall reaction on known concentrations of m-aminobenzoic acid. 

#### 2.9.2. β-Glucuronidase Assay

Fresh cecal and fecal samples were thawed in cold potassium phosphate buffer (0.1 M) having pH 7.0. The cecal and fecal suspensions were homogenized in a pre-chilled homogenizer. The filtrate was sonicated for 30 s (six times) bursts at 4 °C and then supernatant was collected by centrifuging at 500× *g* for 15 min. The enzyme reaction was carried out using supernatant at 37 °C (pH 6.8), 500 µL was the total volume of reaction mixture containing 0.02 M potassium phosphate buffer, 0.1 mM EDTA, 1 mM phenolphthalein-β-d-glucuronide, and 50 µL cecal and fecal extracts. At the end of reaction 2.5 mL glycine buffer (0.2 M) having pH 10.4 containing NaCl (0.2 M) was added to stop the reaction. A standard curve of phenolphthalein was prepared for comparison to determine the amount of phenolphthalein released, all readings were taken at 540 nm.

#### 2.9.3. β-Glucosidase Assay

The samples for β-glucosidase assay were prepared as described for the β-glucuronidase assay. Reaction was carried out at 37 °C (pH 7.0), 500 µL was the total volume of reaction mixture containing 0.1 M potassium phosphate buffer, 1 mM nitrophenyl-β-d-glucoside and 100 µL cecal and fecal extracts. At the end of reaction 2.5 mL sodium hydroxide of 0.01 M concentration was added in reaction mixture to stop chemical process. A standard curve of nitrophenol was prepared for comparison to determine the amount of nitrophenol released, all readings were taken at 420 nm.

### 2.10. Short Chain Fatty Acids

After collection, cecal and fecal contents were stored at −80 °C until analysis of SCFAs using gas chromatography (Agilent 6890 Plus gas chromatograph, Santa Clara, CA, USA) and expressed as µmol/g of cecal/fecal material [29]. One gram of cecal/fecal sample was thawed and suspended in 5 mL of distilled water followed by homogenization (UltraTurrax T 25, Staufen, Germany) for 3 min, resulting in a 20% (*w*/*v*) cecal/fecal suspension. The HCl (5 M) was used to adjust the pH of suspension to 2–3 and was placed on shaker for 10 min at room temperature followed by centrifugation (5000 rpm) for 20 min and the clear supernatant was separated. 2-ethylbutyric acid solution was added in supernatant as internal standard having final concentration of 1 mM and this prepared supernatant was used for the quantification of acetic, propionic, and butyric acids using standards of these fatty acids.

### 2.11. Statistical Analysis

All of the results were expressed as mean ± standard error (SE). The inter group variation was assessed by one way analysis of variance (ANOVA) using SPSS software (ver. 18). In all significant results, post hoc comparison was performed using the Duncan Multiple Range test (DMRt). Differences were considered significant at *p* < 0.05.

## 3. Results

### 3.1. Body Weight and Food Intake

All groups were provided with a basal diet, along with DMH and different treatments of prebiotics, GOS, and inulin, except group G1 (fed only on basal diet) and G2 (basal diet and DMH without prebiotics). There were no significant differences in food intake among all groups (Table 1).

**Figure 1:**
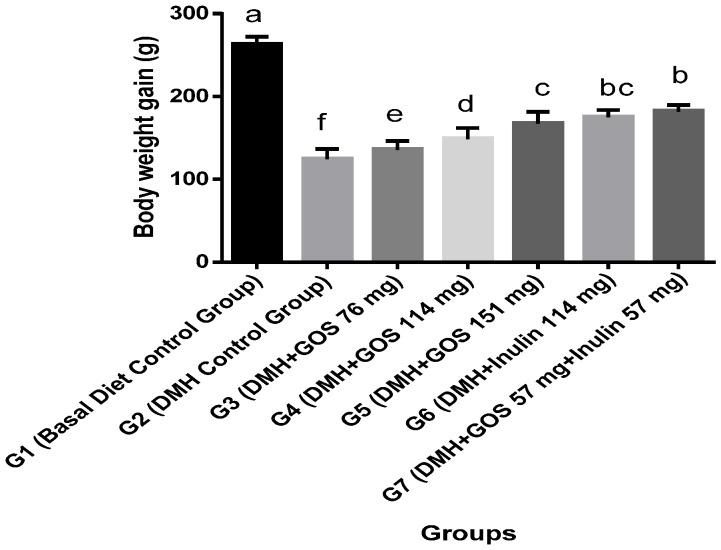
Change in body weights in DMH-initiated and non-initiated animals. Among DMH-treated animals, group G7 attained highest body weight. Body weight loss was maximum in the DMH control group. Values are expressed as mean ± SE. Bars with no letters in common are significantly different (*p* ≤ 0.05).

Group G1 gained significant body weight (*p* ≤ 0.05) as compared to all other groups. DMH administration significantly reduced the body weight compared to group G1 as shown in Figure 1 (Body weight gain = final body wt. − initial body wt.). Group G2 gained the lowest body weight as compared to all other groups. Groups G3–G5, given GOS treatments, showed resistance to DMH-induced body weight loss and manifested dose-dependent alleviation of their body weights (*p* ≤ 0.05) as compared to Group G2. Moreover, inulin, at a dose of 114 mg, exerted a better effect on body weight recovery than GOS treatment at the same dose. Interestingly, the combination of GOS and inulin achieved the best results in preventing DMH-induced body weight loss (Figure 1).

### 3.2. Individual Effect of GOS and Inulin on Aberrant Crypt Foci (ACF)

**Figure 2:**
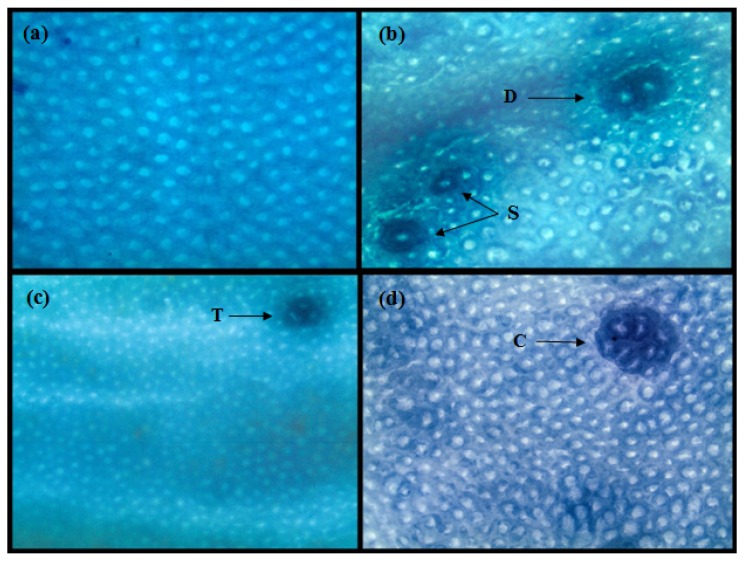
Histological examination of colon for ACF. (**a**) Normal crypts of control group animals; (**b**–**d**) DMH-treated animals showing aberrant crypt foci. Arrows indicate ACF: singlet (S); doublet (D); triplet (T); and cluster (C).

ACF analysis was carried out at the end of 16 weeks and results are shown in Table 2 and Figure 2. The effect of prebiotics on the occurrence and distribution of ACF among different parts of the colon is shown in Table 2. There was no ACF detected in basal diet control group (G1). With DMH treatment, total numbers of ACF in the colon in Group G2 were 170.4 ± 7.34. A prominent reduction in the numbers of ACF was observed in DMH + prebiotic treated animals as compared to Group G2. Groups G4 and G5, given GOS, showed significant (*p* ≤ 0.05) reductions in total ACF as compared to G2. Group G5 saw a significant (*p* ≤ 0.05) reduction of total ACF as compared to other GOS groups (G3 and G4). Group G6 also showed a significant (*p* ≤ 0.05) reduction of total ACF as compared to G2, G3, and G4, however, it was statistically similar to G5. The maximum percentage of total ACF inhibition was achieved in G6 (51.2%) in case of individual effects of prebiotics, followed by G5 (41.4%) and G4 (22.8%).

### 3.3. Combined Effect of Inulin and GOS on ACF

The combined effects of GOS and inulin showed much better results in ACF inhibition than their individual treatments. Group G7 showed a significant (*p* ≤ 0.05) reduction of ACF in all parts of colon (proximal, middle and distal). The combination of GOS and inulin treatment showed 55.8% inhibition of ACF formation in animals, which is significantly (*p* ≤ 0.05) higher than the GOS treatment alone.

### 3.4. SCFA, pH, and Ammonia

Groups G3–G5, which were receiving GOS, showed significant (*p* ≤ 0.05) increases in cecum acetate levels as compared to Group G2, while groups G4 and G5 were higher (*p* ≤ 0.05) in cecum butyrate, fecal acetate, and propionate levels, only G5 among GOS groups showed significant (*p* ≤ 0.05) higher concentrations of fecal butyrate compared to the DMH control (G2) group, as shown in Table 3. Inulin (G6) treatment showed significantly higher levels (*p* ≤ 0.05) of cecum and fecal acetate, propionate, and butyrate concentrations when compared with the DMH control group (G2). Interestingly, the combination of GOS and inulin treatment (G7) exhibited the highest cecum and fecal levels of acetate, propionate, and butyrate among all of the treatment groups. In addition to SCFA, pH and ammonia levels are also indicators of fermentation status. The fecal and cecal pH was not significantly altered among all groups (Table 3). Nevertheless, prebiotic treatments significantly reduced (*p* ≤ 0.05) the ammonia levels in cecum and fecal overall. The combination of GOS and inulin significantly (*p* ≤ 0.05) reduced the levels of cecal and fecal ammonia than GOS groups alone at doses of 114 mg and 76 mg, respectively.

### 3.5. Bacterial Enzymes

Overall, Group G2 showed higher levels of enzyme activities in cecum and fecal contents. Among those groups which were given GOS as treatments, G5 showed significantly (*p* ≤ 0.05) lower concentrations of β-glucoronidase, and both G4 and G5 showed significantly (*p* ≤ 0.05) lower concentrations of nitroreductase and azoreductase in cecum contents compared to G2. Regarding fecal content, G4 and G5 were able to significantly (*p* ≤ 0.05) reduce the concentrations of β-glucoronidase, nitroreductase, and azoreductase, while only G5 was able to reduce (*p* ≤ 0.05) the concentration of β-glucosidase compared to G2. Group G6, which was given inulin, also showed significantly (*p* ≤ 0.05) lower concentrations of all enzymes in cecum and fecal contents as compared to DMH control group (G2). We observed that combination treatment of GOS and inulin (G7) resulted in the lowest concentrations of all enzymes in cecal and fecal samples, as shown in Table 4. Specifically, addition of a GOS and inulin mixture in both cecum and feces reduced the activity of all enzymes more efficiently, compared to individual treatments of GOS and inulin.

## 4. Discussion

CRC is one of the most common cancers and is the leading cause for morbidity and mortality, even in developed countries. Scientists have focused on various bioactive components in foods to evaluate their role in prevention of CRC. Our data demonstrate that the combination of GOS and inulin provided a significant preventive effect against the development of CRC. Furthermore, the inhibition of ACF formation was significantly higher using this novel combination than inulin and GOS treatments alone. The results of current study also suggest that, in comparison to GOS or inulin individually in different doses, the combination of GOS and inulin rendered better resistance against DMH-induced body weight loss and showed higher levels of cecal and fecal SCFA (acetate, propionate, and butyrate).

ACF are pre-adenomatous morphological putative lesions within the colonic mucosa that may lead to progression to colon cancer. In the present study colon samples were collected after 16 weeks for ACF analysis and, as reported in previous literature, this time period is sufficient for the development of ACF and to observe the effects of treatments. Our results indicated that various doses of prebiotics significantly (*p* > 0.05) inhibited DMH-induced colonic ACF formation in rats. We also showed that GOS inhibited ACF formation in rats in a dose-dependent manner; among all GOS groups, G5 had the maximum effect on ACF inhibition (55.8%), followed by G4 (41.4%) and G3 (22.8%) when compared to the G2 group. Only one previous study [20] reported a high dose of commercial GOS (β-1,4 linkage), at 20% concentration in the diet, inhibited ACF formation. Interestingly, our results showed that the combination of prebiotics with both GOS and inulin (G7) had not only reduced the number of ACF in the proximal, middle, and distal colon, but also altered the distribution of ACF in the entire colon (Table 2). This suggests that administration of prebiotics in combination is able to exert a pronounced chemopreventive effect on preneoplastic ACF formation. Previous experimental studies also support our findings and suggest that prebiotics are effective enough to inhibit total ACF counts chemically-induced in the colon [8,20,30,31,32]. As it is observed that inulin was better than GOS in reducing ACF counts, it might be due to inulin being slowly degraded and passing further along the colon before being completely degraded in the upper parts of intestine [33]. Another reason might be the monosaccharides in GOS which promote the growth of microbes without discriminating beneficial and harmful microbes. A previous study reported that inulin was able to inhibit 78.8% ACF formation in rats [30]. The reduced ACF counts in prebiotic-treated animals may be due to the modulation of microbiota and increased concentrations of SCFAs, as these are important for normal development of colonic epithelial cells [16,31]. 

Those groups which were receiving prebiotics, particularly G5–G7, showed higher levels of SCFA production and reductions in ammonia in cecal and fecal contents of rats (Table 3). The SCFA, especially acetate, propionate, and butyrate, are the major products of prebiotic anaerobic fermentation, which results in the lowering of colon pH and increasing the growth of beneficial bacteria, and ultimately attribute to the health benefits of prebiotics [34]. Among these SCFA, butyrate has received much attention in colon cancer prevention with multiple roles, including intestinal barrier function, minerals absorption, cell growth and differentiation [35], as well as its immuno-modulatory activity through its histone deacetylase (HDAC) inhibitory activity on nuclear factor κB (NF-κB) and its expression in colon carcinoma cells [36]. The butyrigenic effect of prebiotic GOS and inulin is very much desirable and has been suggested to be a major contributor to prevent/inhibit colon carcinogenesis [37]. Higher dietary protein can raise ammonia levels in the colon [38] and increase CRC risk through enhancing colonocyte proliferation [26]. Prebiotic supplementation or increased levels of butyrate in the colon may reduce ammonia-mediated toxicity [39,40]. In the present study, significant (*p* > 0.05) reduction in ammonia and increase in SCFA’s production in cecum and fecal samples (Table 3) during prebiotic treatments indicated a degree of shift from proteolytic activity to saccharolytic fermentation. This is particularly important in the distal colon where proteolytic fermentation predominates, leading to the accumulation of toxic metabolites. A previous study using prebiotic fructans was consistent with our results with lower levels of ammonia in the prebiotic-treated groups [8,40]. In our findings, high concentrations of dietary GOS (G5 151 mg, HED 8 g), inulin (G6 114 mg, HED 6 g), and GOS + inulin (G7, 57 mg each, HED 6 g) significantly (*p* > 0.05) enhanced the production of SCFA with reduction of ammonia and, interestingly, without a change in pH. Similar findings have been observed in earlier studies using prebiotics and probiotics in combination and ITF against CRC [8,16,32]. 

The gastrointestinal tract (GIT), particularly the colon, is considered a complex natural ecosystem occupied by large number of micro-organisms. This microbiota is known to play a key role in the well-being and health of the host [41]. The substances produced by colonic microflora have different effects on the host, such as carcinogenic, genotoxic, tumor-promoting, and anti-carcinogenic activities [15]. Enzymes, particularly β-glucuronidase, β-glucosidase, azoreductase, nitroreductase, 7-β-dehydrogenase, and 7-β-dehydrolase, are involved in conversions of endogenous toxins and genotoxic compounds [34]. It is proven that changes in concentrations of these bacterial enzymes is an indication of change in GIT microbiota [42] In our findings, the levels of enzymes (β-glucuronidase, β-glucosidase, azoreductase and nitroreductase) in cecal and fecal contents were lower in treatment groups. It is well documented that the higher levels of β-glucuronidase and β-glucosidase are considered as biomarkers of colon cancer playing a significant role in colon carcinogenesis and are involved in the carcinogenesis of neoplasms [28], as β-glucuronidase is involved in restoration of the toxic properties of some xenobiotics in colon, its reduced level is taken as a positive effect for colon cancer prevention [43]. Higher levels of bacterial nitroreductase are considered harmful as it is involved in the synthesis of nitrosamines in the colon produced by the interaction of amines with the product of nitrate/nitrite reduction [43]. The decreased activity of β-glucosidase and β-glucuronidase in the present study with prebiotic treatment may be due to the improved intestinal microbiota and many other in vivo factors like pH, ammonia production and substrate availability. Moreover, earlier in vivo studies have also documented that inulin and oligofructose supplementation in fecal bacterial cultures led to enhanced growth of bifidobacterium, having low β-glucuronidase and β-glucosidase activity, and reduced growth of *Escherichia coli* and *Clostridium* sp. that are known to have higher β-glucuronidase activity [30]. The previous study [30] using inulin and lactulose prebiotics also found significantly (*p* ≤ 0.05) decreased activity of β-glucuronidase and β-glucosidase in animals belonging to the inulin + DMH group compared with the lactulose + DMH-treated group strengthens our findings.

## 5. Conclusions

In conclusion, this study reveals that GOS prebiotic treatment has a dose-dependent chemo-preventive effect against DMH-induced ACF formation and CRC-related bio-markers. A GOS dose of 8 g (HED) per day is more effective at producing these outcomes than lower doses of 4 and 6 g per day. Novelty of the present study is utilization of GOS containing β-1,6 and β-1,3 glycosidic linkages, which have never been used before in any cancer prevention study. A novel combination of GOS and inulin exhibited stronger preventive activity than their individual treatments alone. As epidemiological data indicates increasing prevalence of CRC throughout the world, and dietary patterns are considered one of major risk factors, it time to focus on dietary strategies for chemoprevention of CRC. Results of the present study encourage inclusion of prebiotic combinations in routine diet and can be a promising strategy for CRC chemoprevention. Further studies are needed to understand the mechanisms of action of different prebiotics containing a variety of linkages in CRC prevention.

## Figures and Tables

**Table 1:** Average food intake in different groups of animals throughout the experiment.

Groups	Food Intake (g/Rat/Day)
G1 (Basal Diet Control Group)	20.0 ± 0.5 ^a^
G2 (DMH Control Group)	20.6 ± 0.6 ^a^
G3 (DMH + GOS 76 mg)	21.0 ± 0.6 ^a^
G4 (DMH + GOS 114 mg)	20.3 ± 0.5 ^a^
G5 (DMH + GOS 151 mg)	20.1 ± 0.5 ^a^
G6 (DMH + Inulin 114 mg)	21.0 ± 0.6 ^a^
G7 (DMH + GOS 57 mg + Inulin 57 mg)	20.7 ± 0.6 ^a^

Note: Values are expressed as mean ± SE. Means with the same letters are not significantly different (*p* ≤ 0.05).

**Table 2:** Effect of prebiotic treatments on aberrant crypt foci (ACF) in proximal, middle, and distal colon in DMH-initiated and non-initiated animals (*n* = 12).

ACF	G1 (Basal Diet Control Group)	G2 (DMH Control Group)	G3 (DMH + GOS 76 mg)	G4 (DMH + GOS 114 mg)	G5 (DMH + GOS 151 mg)	G6 (DMH + Inulin 114 mg)	G7 (DMH + GOS 57 mg + Inulin 57 mg)
ACF/proximal colon	ND	24.6 ± 2.73 ^a^	23.8 ± 2.87 ^a^	12.6 ± 2.13 ^b^	8.7 ± 1.47 ^b,c^	7.5 ± 1.05 ^b,c^	5.3 ± 0.63 ^c^
ACF/middle colon	ND	39.6 ± 4.03 ^a^	33.3 ± 3.09 ^a^	23.8 ± 2.82 ^b^	15.2 ± 1. 86 ^c^	16.2 ± 1.92 ^b,c^	18.8 ± 2.72 ^b,c^
ACF/distal colon	ND	106.3 ± 6.77 ^a^	92.3 ± 5.50 ^a^	95.2 ± 5.63 ^a^	76.0 ± 5.32 ^b^	59.6 ± 4.98 ^c^	51.2 ± 4.14 ^c^
Total ACF/colon	ND	170.4 ± 7.34 ^a^	153.1 ± 9.23 ^a^	131.6 ± 9.77 ^b^	99.8 ± 8.93 ^c^	83.3 ± 5.52 ^c,d^	75.3 ± 6.95 ^d^
% of total ACF inhibition	-	-	10.2	22.8	41.4	51.2	55.8

Note: Values are expressed as m ± SE. Means in the same row with different superscript letters are significantly different (*p* ≤ 0.05); DMH = 1,2 dimethylhydrazine dihydrochloride (4 × 40 mg/kg body weight, subcutaneous); ND = Not detected.

**Table 3:** Effect of prebiotic treatments on SCFA, pH, and ammonia concentrations of cecal and fecal contents in DMH-initiated and non-initiated animals (*n* = 12).

Parameters		G1 (Basal Diet Control Group)	G2 (DMH Control Group)	G3 (DMH + GOS 76 mg)	G4 (DMH + GOS 114 mg)	G5 (DMH + GOS 151 mg)	G6 (DMH + Inulin 114 mg)	G7 (DMH + GOS 57 mg + Inulin 57 mg)
CECUM	Acetate	82.6 ± 2.73 ^c,d^	80.5 ± 3.30 ^e^	86.8 ± 3.66 ^c,d^	91.4 ± 3.08 ^b,c^	97.1 ± 2.89 ^a,b^	99.3 ± 3.55 ^a,b^	104.0 ± 3.95 ^a^
	Propionate	23.3 ± 1.89 ^c^	22.7 ± 1.96 ^c^	24.7 ± 2.21 ^b,c^	27.8 ± 1.41 ^b,c^	28.2 ± 1.54 ^b,c^	29.3 ± 1.58 ^a,b^	33.6 ± 1.79 ^a^
	Butyrate	15.7 ± 0.79 ^c,d^	15.4 ± 1.08 ^d^	16.3 ± 1.13 ^c,d^	18.8 ± 1.08 ^b,c^	19.7 ± 1.19 ^a,b^	20.2 ± 0.99 ^a,b^	22.7 ± 1.44 ^a^
	pH	6.6 ± 0.15	6.7 ± 0.14	6.5 ± 0.12	6.5 ± 0.07	6.4 ± 0.11	6.4 ± 0.13	6.3 ± 0.18
	Ammonia	15.3 ± 1.48 ^a^	15.6 ± 1.69 ^a^	14.9 ± 2.05 ^a^	12.8 ± 1.62 ^a,b^	11.1 ± 1.06 ^a,b,c^	10.3 ± 1.15 ^b,c^	7.7 ± 0.64 ^c^
FECAL	Acetate	55.4 ± 2.60 ^c^	53.8 ± 2.84 ^c^	57.5 ± 3.08 ^b,c^	63.7 ± 2.38 ^a,b^	67.1 ± 3.16 ^a^	69.6 ± 2.58 ^a^	70.4 ± 2.64 ^a^
	Propionate	15.4 ± 0.83 ^b,c^	15.3 ± 0.78 ^c^	16.8 ± 0.98 ^b,c^	18.4 ± 1.05 ^b^	21.3 ± 1.04 ^a^	22.3 ± 1.07 ^a^	24.3 ± 1.27 ^a^
	Butyrate	5.5 ± 0.51 ^c^	5.3 ± 0.53 ^c^	6.2 ± 0.67 ^c^	7.3 ± 0.66 ^b,c^	8.4 ± 0.73 ^a,b^	8.8 ± 0.73 ^a,b^	9.8 ± 0.94 ^a^
	pH	6.6 ± 0.16	6.6 ± 0.14	6.5 ± 0.10	6.4 ± 0.08	6.4 ± 0.11	6.4 ± 0.13	6.3 ± 0.11
	Ammonia	10.1 ± 1.13 ^a^	10.6 ± 1.34 ^a^	8.8 ± 0.84 ^a,b^	6.8 ± 0.87 ^b,c^	5.9 ± 0.95 ^b,c^	5.5 ± 0.92 ^c^	4.2 ± 0.37 ^c^

Note: Values are expressed as mean ± SE. Means in the same row with different letters are significantly different (*p* ≤ 0.05); DMH = 1,2 dimethylhydrazine dihydrochloride (4 × 40 mg/kg body weight, subcutaneous); SCFAs = μmol/g; ammonia = mM.

**Table 4:** Effect of prebiotic treatments on cecal and fecal enzyme activities in DMH-initiated and non-initiated animals (*n* = 12).

Enzymes		G1 (Basal Diet Control Group)	G2 (DMH Control Group)	G3 (DMH + GOS 76 mg)	G4 (DMH + GOS 114 mg)	G5 (DMH + GOS 151 mg)	G6 (DMH + Inulin 114 mg)	G7 (DMH + GOS 57 mg + Inulin 57 mg)
CECUM	β-Glucosidase	0.97 ± 0.12 ^a,b^	1.13 ± 0.21 ^a^	1.09 ± 0.11 ^a^	0.83 ± 0.11 ^a,b,c^	0.77 ± 0.11 ^a,b,c^	0.64 ± 0.12 ^b,c^	0.52 ± 0.13 ^c^
	β-Glucoronidase	3.15 ± 0.19 ^a^	3.19 ± 0.19 ^a^	2.94 ± 0.18 ^a^	2.57 ± 0.18 ^a,b^	2.19 ± 0.27 ^b^	2.27 ± 0.25 ^b^	1.94 ± 0.19 ^b^
	Nitroreductase	4.17 ± 0.65 ^a^	4.33 ± 0.53 ^a^	4.16 ± 0.82 ^a^	2.58 ± 0.54 ^b^	2.17 ± 0.27 ^b^	2.02 ± 0.21 ^b^	1.68 ± 0.19 ^b^
	Azoreductase	10.75 ± 1.14 ^a^	10.58 ± 1.06 ^a^	8.42 ± 0.97 ^a,b^	7.25 ± 0.93 ^b^	5.67 ± 0.69 ^b,c^	6.08 ± 0.86 ^b,c^	4.33 ± 0.58 ^c^
FECAL	β-Glucosidase	0.80 ± 0.12 ^a,b^	1.12 ± 0.13 ^a^	0.73 ± 0.08 ^b,c^	0.81 ± 0.10 ^a,b^	0.57 ± 0.10 ^b,c^	0.47 ± 0.10 ^b,c^	0.43 ± 0.11 ^c^
	β-Glucoronidase	2.56 ± 0.33 ^a^	2.64 ± 0.35 ^a^	2.14 ± 0.26 ^a,b^	1.71 ± 0.19 ^b,c^	1.50 ± 0.25 ^b,c^	1.37 ± 0.12 ^c^	1.17 ± 0.11 ^c^
	Nitroreductase	2.67 ± 0.36 ^a,b^	2.92 ± 0.45 ^a^	2.66 ± 0.62 ^a,b^	1.75 ± 0.18 ^b,c^	1.27 ± 0.16 ^c^	1.14 ± 0.17 ^c^	0.97 ± 0.16 ^c^
	Azoreductase	6.58 ± 0.85 ^a^	6.83 ± 0.81 ^a^	5.75 ± 0.79 ^a,b^	4.33 ± 0.54 ^b,c^	3.67 ± 0.33 ^c,d^	3.42 ± 0.23 ^c,d^	2.17 ± 0.28 ^d^

Note: Values are expressed as mean ± SE. Means in the same row with different letters are significantly different (*p* ≤ 0.05); DMH = 1,2 dimethylhydrazine dihydrochloride (4 × 40 mg/kg body weight, subcutaneous); β-Glucosidase = μg/min/mg cecal or fecal protein; β-Glucoronidase = μg/min/mg cecal or fecal protein; Nitroreductase = μg/h/mg cecal or fecal protein; Azoreductase = μg/h/mg cecal or fecal protein.
